# Imagined Speech Classification Using EEG and Deep Learning

**DOI:** 10.3390/bioengineering10060649

**Published:** 2023-05-26

**Authors:** Mokhles M. Abdulghani, Wilbur L. Walters, Khalid H. Abed

**Affiliations:** Department of Electrical & Computer Engineering and Computer Science, College of Sciences, Engineering & Technology, Jackson State University, Jackson, MS 39217, USA; mokhles.m.abdulghani@students.jsums.edu (M.M.A.); wilbur.l.walters@jsums.edu (W.L.W.)

**Keywords:** inner speech, imagined speech, EEG decoding, brain–computer interface (BCI), LSTM, wavelet scattering transformation (WST)

## Abstract

In this paper, we propose an imagined speech-based brain wave pattern recognition using deep learning. Multiple features were extracted concurrently from eight-channel electroencephalography (EEG) signals. To obtain classifiable EEG data with fewer sensors, we placed the EEG sensors on carefully selected spots on the scalp. To decrease the dimensions and complexity of the EEG dataset and to avoid overfitting during the deep learning algorithm, we utilized the wavelet scattering transformation. A low-cost 8-channel EEG headset was used with MATLAB 2023a to acquire the EEG data. The long-short term memory recurrent neural network (LSTM-RNN) was used to decode the identified EEG signals into four audio commands: up, down, left, and right. Wavelet scattering transformation was applied to extract the most stable features by passing the EEG dataset through a series of filtration processes. Filtration was implemented for each individual command in the EEG datasets. The proposed imagined speech-based brain wave pattern recognition approach achieved a 92.50% overall classification accuracy. This accuracy is promising for designing a trustworthy imagined speech-based brain–computer interface (BCI) future real-time systems. For better evaluation of the classification performance, other metrics were considered, and we obtained 92.74%, 92.50%, and 92.62% for precision, recall, and F1-score, respectively.

## 1. Introduction

An enormous body of research has been conducted over the past decade aiming to convert human brain signals to speech. Although experiments have shown that the excitation of the central motor cortex is elevated when visual and auditory cues are employed, the functional benefit of such a method is limited [1]. Imagined speech, sometimes called inner speech, is an excellent choice for decoding human thinking using the brain–computer interface (BCI) concept. BCI is being developed to progressively allow paralyzed patients to interact directly with their environment. Brain signals usable with the BCI systems can be recorded with a variety of common recording technologies, such as magnetoencephalography (MEG), electrocorticography (ECOG), functional magnetic resonance imaging (fMRI), functional near-infrared spectroscopy (fNIRS), and electroencephalography (EEG). EEG headsets are used to record the electrical activities of the human brain. EEG-based BCI systems can convert the electrical activities of the human brain into commands. An EEG-based implementation is considered an effective way to help patients with a high level of disability or physical challenges control their supporting systems, such as wheelchairs, computers, or wearable devices [2,3,4,5]. Moreover, in our very recent research [6,7], we accomplished excellent accuracy in classifying EEG signals to control a drone, and considered the Internet of Things (IoT) to design an Internet of Brain-Controlled Things (IoBCT) system.

Applying soft-computing tools, such as artificial neural networks (ANNs), genetic algorithms, and fuzzy logic helps designers implement intelligent devices that fit the needs of physically challenged people [8]. Siswoyo et al. [9] suggested a three-layer neural network to develop the mapping from input received from EEG sensors to three control commands. Fattouh et al. [10] recommended a BCI control system to distinguish between four control commands alongside the emotional status of the user. If the user is satisfied, the specific control command is still executed, otherwise the controller should stop the implementation and ask the patient to choose another command. Decoding the brain waves and presenting them as an audio command is a more reliable solution to avoid the execution of unwanted commands, and this is mainly true if the user can listen to the translated commands from their brain and confirm or deny the execution of that command. A deep learning algorithm offers a valuable solution for processing, analyzing, and classifying brainwaves [11]. Modern studies have concentrated, in both healthy individuals and physically challenged patients, primarily on communicating their thoughts [12]. Vezard et al. [13] reached a 71.6% accuracy in a binary alertness states (BAS) estimation by applying the common spatial pattern (CSP) to extract the feature. The methods in [14,15] were used to discriminate between different motor imagery tasks and reached an EEG classification accuracy of just 54.6% and 56.76%, respectively. This was achieved by applying a multi-stage CSP for the EEG dataset feature extraction. In [16], researchers employed the power of a deep learning algorithm using the recurrent neural network (RNN) to process and classify the EEG dataset.

For cheaper and more easily set up and maintained BCI systems, it is preferable to have as few EEG channels as possible. There are two types of BCI systems: online systems, such as those described in [17,18], and offline BCI systems, such as the systems described in [19]. In the offline EEG system, the EEG data recorded from the participants are stored and processed later; on the other hand, the online BCI system processes the data in real time, such as in the case of a moving wheelchair. Recent research [20] revealed that EEG-based inner speech classification accuracy can be improved when auditory cues are used. Wang et al. [21] demonstrated in their study, which was based on common spatial patterns and event-related spectral perturbation (ERSP), that the highly significant EEG channels for classifying inner speech are the ones laid on the Broca’s and Wernicke’s regions. Essentially, the Wernicke region is responsible for ensuring that the speech makes sense, while the Broca region ensures that the speech is produced fluently. Given that both Wernicke’s and Broca’s regions participate in inner speech, it is not easy to eliminate the effect of the auditory activities from the EEG signal recorded during speech imagination. Indeed, some researchers have suggested that auditory and visual activities are essential to decide the brain response [22,23].

In most studies, the participants are directed to imagine speaking the commands only once. However, in [24,25], the participants must imagine saying a specific command multiple times in the same recording. In [26], the commands “left”, “right”, “up” and “down” were used. This choice of commands is not only motivated by the suitability of these commands in practical applications but also because of their various manner and places of articulation. Maximum classification accuracies of 49.77% and 85.57% were obtained, respectively. This was accomplished using the kernel extreme learning machine (ELM) classification algorithm. Significant efforts have been recently published by Nature [27], where a 128-channel EEG headset was used to record inner speech-based brain activities. The acquired dataset consists of EEG signals from 10 participants recorded by 128 channels distributed all over the scalp according to the ‘ABC’ layout of the manufacturer of the EEG headset used in this study. The participants were instructed to produce inner speech for four words: ‘up’, ‘down’, ‘left’, and ‘right’, based on a visual cue they saw in each trial. The cue was an arrow on a computer screen that rotated in the corresponding directions. This was repeated 220 times for each participant. However, since some participants reported fatigue, the final number of trials included in the dataset for each participant differed slightly. The total number of trials was 2236, with an equal number of trials per class for all participants. The EEG signals included event markers and were already preprocessed. The preprocessing included a bandpass filter between 0.5–100 Hz, a notch filter at 50 Hz, artifact rejection using independent component analysis (ICA), and down-sampling to 254 Hz. The long-short term memory (LSTM) algorithm was used in [28,29] to classify EEG signals. In [28], 84% accuracy in EEG data classification was achieved. In [29], an excellent accuracy of 98% was achieved in classifying the EEG-based inner speech, but researchers used an expensive EEG headset. Obtaining high accuracy in classifying brain signals is considered essential in the design of future brain-controlled systems, which can be tested in real-time or in simulation software such as V-Rep [30] to check for any uncounted errors.

Most researchers have used high-cost EEG headsets to build BCI systems for imagined speech processing. Using the RNN for time-series input showed good execution in extracting features over time, and achieved an 85% classification accuracy. Although innovative techniques in conventional representations, such as event-related potential (ERP), and steady-state visual evoked potential (SSVEP), have expanded the communication ability of patients with a high level of disability, these representations are restricted in their use for the availability of a visual stimulus [31,32]. Practical research studied imagined speech in EEG-based BCI systems and showed that imagined speech could be extrapolated using texts with high discriminatory pronunciation [33]. Hence, BCI-based gear can be controlled by processing brain signals and extrapolating inner speech [34]. Extensive research has been conducted to develop BCI systems using inner speech and motor imagery [35]. To investigate the feasibility of using EEG signals for imagined speech recognition, a research study reported promising results on imagined speech classification [36]. In addition, a similar research study examined the feasibility of using EEG signals for inner speech recognition and increasing the efficiency of such use [37].

In this paper, we have used a low-cost low-channel 8-channel EEG headset, g.tec Unicorn Hybrid Black+ [38], with MATLAB 2023a for recording the dataset to decrease the computational complexity required later in the processing. Then, we decoded the identified signals into four audio commands: up, down, left, and right. These commands were performed as an imagined speech by four healthy subjects whose ages are between 20 and 56 years old, including two females and two males. The EEG signals were recorded while the imagination of speech occurred. An imagined speech-based BCI model was designed using deep learning. Audio cues were used to stimulate the motor imagery of the participants in this study, and the participants responded with imagined speech commands. Pre-processing and filtration techniques were employed to simplify the recorded EEG dataset and speed up the learning process of the designed algorithm. Moreover, the short-long term memory technique was used to classify the imagined speech-based EEG dataset.

## 2. Materials and Methods

We considered research methodologies and equipment in order to optimize the system design, simulation, and verification.

### 2.1. Apparatus

In order to optimize the system design, reduce the cost of the designed system, and decrease the computational complexity, we used a low-cost EEG headset. We used a low number of EEG channels with the focus instead on the placement of EEG sensors at the proper locations on the scalp to measure specific brain activities. The EEG signals were recorded using the g.tec Unicorn Hybrid Black+ headset. It has eight-channel EEG electrodes with a 250 Hz sampling frequency. It records up to seventeen channels, including the 8-channel EEG, a 3-dimensional accelerometer, a gyro, a counter signal, a battery signal, and a validation signal. The EEG electrodes of this headset are made of a conductive rubber that allows recording dry or with gel. Eight channels were recorded on the following positions: (FZ, C3, CZ, C4, PZ, PO7, OZ, and PO8) which are the standard electrodes positions of g.tec Unicorn Hybrid Black+ headset. The used g.tec headset provides standard EEG head caps of various sizes with customized electrode positions. A cap of appropriate size was chosen for each participant by measuring the head boundary with a soft measuring tape. All EEG electrodes were placed in the marked positions in the cap, and the gap between the scalp and the electrodes was filled with a conductive gel provided by the EEG headset manufacturer.

**Figure 1:**
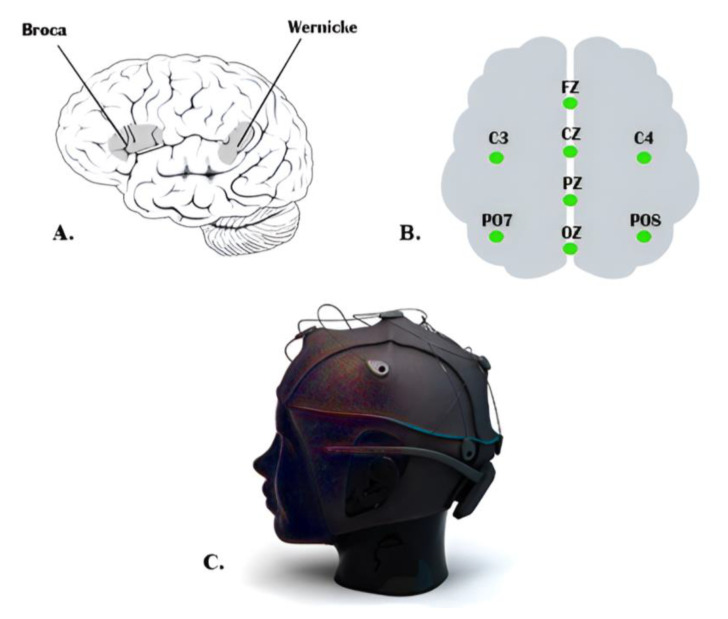
(**A**) Broca’s and Wernicke’s regions, (**B**) The electrode positions of the system. Ground and reference are fixed on the back of ears (mastoids) with a disposable sticker, (**C**) 8-channel EEG headset.

We considered the international electrode placement 10-20 recommended by the American clinical neurophysiology society [39]. The head cap was adjusted to ensure their electrodes were placed as close to Broca’s and Wernicke’s regions as possible, which we assumed to produce good quality imagined speech-based EEG signals. Figure 1 shows the g.tec Unicorn Hybrid Black+ headset with the electrode map. Ground and reference were positioned on the back of the ears (mastoids) of the participant using a disposable sticker.

### 2.2. Procedure and Data Collection

The study was conducted in the Department of Electrical & Computer Engineering and Computer Science at Jackson State University. The experimental protocol was approved by the Institutional Review Board (IRB) at Jackson State University in the state of Mississippi [40]. Four healthy participants, two females and two males in the age range (20–56), with no speech loss, no hearing loss, and with no neurological or movement disorders, participated the experiment and signed their written informed consent. Each participant was a native English speaker. None of the participants had any previous BCI experience and contributed to approximately one hour of recording. In this work, the participants are classified by aliases “sub-01” through “sub-04”. The age, gender, and language information about the participating subjects is provided in Table 1.

The experiment was designed to record the brain’s activities while imagining speaking a specific command. When we usually talk to each other, our reactions will be based on what we hear or sometimes on what we see. Therefore, we could improve the accuracy of classifying different commands by allowing participants to respond to an audio question. Each participant was seated in a comfortable chair in front of another chair where a second participant would announce the question as an audio cue. To familiarize the participant with the experimental procedures, all experiment steps were explained before the experiment date and before signing the consent form. The experimental procedures were explained again during the experiment while the EEG headset and the external electrodes were placed. The setup procedure took approximately 15 min. Four commands were chosen to be imagined as a response to the question: “Where do you want to go?” A hundred recordings were acquired for each command, with each participant completing 25 recordings. Each recording lasted approximately 2 min and required two participants to be present. Unlike the procedure in [24,25], we did not set a specific number for each command to be repeated. When the recording began, the question was announced after 10 to 12 s as audio cues by one of the other three participants. After 10 s, the participant started executing his response for 60 s by continuously imagining saying the required command, and the recording was stopped after 10 s. In each recording, the participant responded by imagining saying the specified command, which was one of the four commands. Since we have four commands, the total recorded EEG dataset for all was 400 recordings.

**Figure 2:**
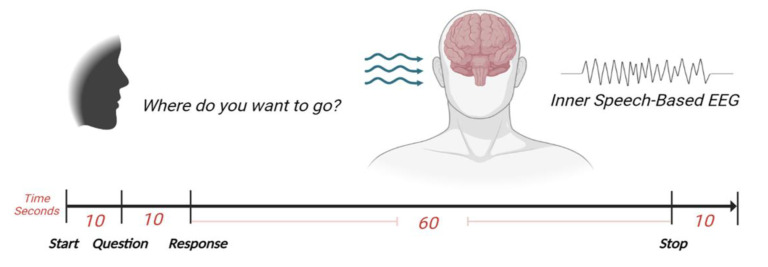
The recording procedure.

**Figure 3:**
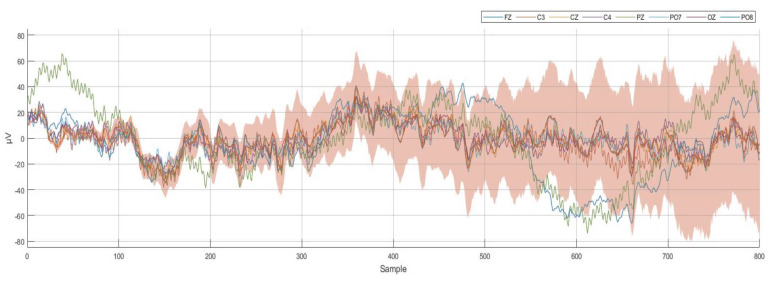
Sample of the recorded 8−channel raw EEG dataset at 250 Hz (250 samples per second).

The recorded EEG dataset for all 400 recordings was labeled and stored; then, the EEG dataset was imported into MATLAB to prepare it for processing. The EEG dataset was processed and classified together without separating them according to their corresponding participants, so that we could evaluate our designed algorithm according to its performance in dealing with a dataset from different subjects. For each command, the first 25 recordings were for subject 1, the second 25 recordings were for subject 2, etc. After finishing the classification process, the results were labeled according to the order of the participant’s dataset. Figure 2 illustrates the recording and signal processing procedures. Figure 3 shows a sample of the recorded 8-channel raw EEG signals.

### 2.3. Data Pre−Processing and Data Normalization

**Figure 4:**
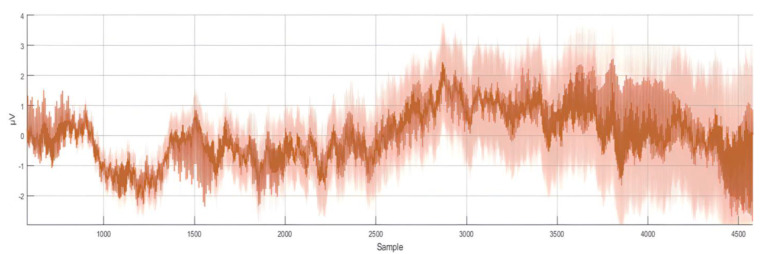
Eight−channel normalized EEG dataset at 250 Hz (250 samples per second).

Preprocessing the raw EEG signals is essential to remove any unwanted artifacts arising from the movement of face muscles during the recording process from the scalp that could affect the accuracy of the classification process. The recorded EEG signals were analyzed using MATLAB, where a bandpass filter between 10 and 100 Hz was used to eliminate any noisy signals from the EEG. This filtering bandwidth maintains the range of frequency bands corresponding to the human brain EEG frequency limit [41]. Then, normalization (vectorization) and feature extraction techniques were applied to simplify the dataset and reduce the computing power required to classify the four commands. The dataset was divided into 320 recordings and 80 recordings for the testing dataset (80% for training and 20% for testing). The EEG dataset was acquired from eight EEG sensors, and contained different frequency bands with different amplitude ranges. Thus, it was beneficial to normalize the EEG dataset to boost the training process speed and get as many accurate results as possible. The training and testing dataset were normalized by determining the mean and standard deviation for each of the eight input signals. Then, the mean value was calculated for both the training and testing dataset. Then, the results for both were divided by the standard deviation as follows:

(1) $$EEG_{Normalized}=\frac{X-\mu }{\sigma }$$

where (*X*) is the raw EEG signal, (*µ*) is the calculated mean value, and (*σ*) is the calculated standard deviation. After the normalization procedure, the dataset was prepared for the training process. Figure 4 shows the normalized representation of the 8-channel raw EEG signals.

### 2.4. Feature Extraction

Wavelet scattering transform is a knowledge-based feature extraction technique that employs complex wavelets to balance the discrimination power and stability of the signal. This technique filters the signal by assembling a cascade of wavelet decomposition coefficients, complex modulus, and lowpass-filtering processes. The wavelet scattering transformation method facilitates the modulus and averaging process of the wavelet coefficients to acquire stable features. Then, the cascaded wavelet transformations are employed to retrieve the high-frequencies data loss that occurred due to the previous wavelet coefficients’ averaging modulus process. The obtained wavelet scattering coefficients retain translation invariance and local stability. In this feature-extracting procedure, a series of signal filtrations was applied to construct a feature vector representing the initial signal. This filtration process continued until the feature vector for the whole signal length was constructed. A feature matrix was constructed for the eight EEG signals. As an outcome of the normalization stage, the obtained dataset consists of one vector with many samples for each command in each of the 100 recordings. Training the deep learning algorithm with a similar dataset is computationally expensive. For instance, in the first recording of the command up, a (1 × 80,480) vector was constructed after the normalization stage. After filtering the dataset for all 100 recordings and using wavelet scattering transformation, eight features were extracted and the (1 × 80,480) vector of the normalized data was minimized to an (8 × 204) matrix for each recording.

**Figure 5:**
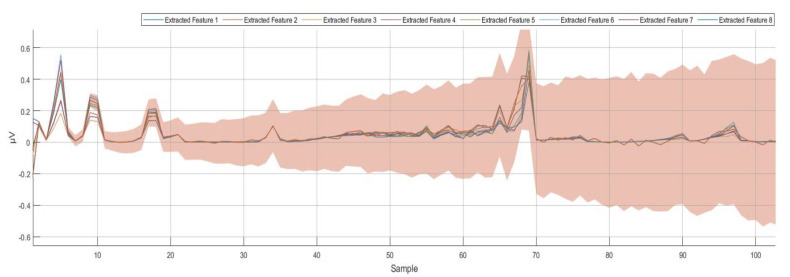
Eight−extracted features using wavelet scattering transformation at 250 Hz (250 samples per second).

Using the wavelet scattering transformation for all the recorded dataset (training and testing datasets) minimized the time spent during the learning process. Moreover, the wavelet scattering transformation provided more organized and recognizable brain activities. Using the wavelet scattering transformation allowed us to optimize the classifications generated by the deep learning algorithm for distinguishing between the four different commands more accurately. Figure 5 shows the eight extracted features after applying the wavelet scattering transformation.

### 2.5. Data Classification

The normalization and feature extraction techniques were used with both the learning and testing datasets to enhance the classification accuracy of the designed BCI system. At this point, the processed datasets were prepared to be trained in deep learning. An LSTM is a type of RNN that can learn long-term dependencies among time steps of a sequenced dataset. The LSTM network has been seen to be more operative than feed-forward neural networks (FNN) and the regular RNN in terms of sequences prediction due to their capability for remembering significant information or values for a long period of time. An LSTM network is frequently used for processing and predicting or classifying sequenced time-series data [42]. A detailed explanation of the LSTM network can be found in [43,44].

The classification model for the recorded EEG dataset was constructed using the LSTM architecture. On the input side, the LSTM was constructed to have an input layer receiving sequence signals, which were eight time-series EEG signals. On the output side, the LSTM was constructed to have a one-vector output layer with rectified liner unit (ReLU) activation function. The output values were set to be (0, 0.5, 0.7, 1.0) for the desired four commands: up, down, left, and right, respectively. During the training process of the used LSTM model, we noticed that limiting the output values of the four indicated classes to between zero and one made the learning faster and more efficient. An AMD Ryzen 7 1700X processor was used for the training, and the training took less than 1 hour with the selected output values, while it took longer than 1.5 h when integer values between 1 to 10 were used. Additionally, having an output value between 0 and 1 offers easier scaling and mapping for the output when the designed algorithm is uploaded to a microcontroller to be tested in real time, especially when one analog output is used for all the output classes. Three LSTM layers were chosen, with 80 hidden units followed by a dropout layer between them. 

**Figure 6:**
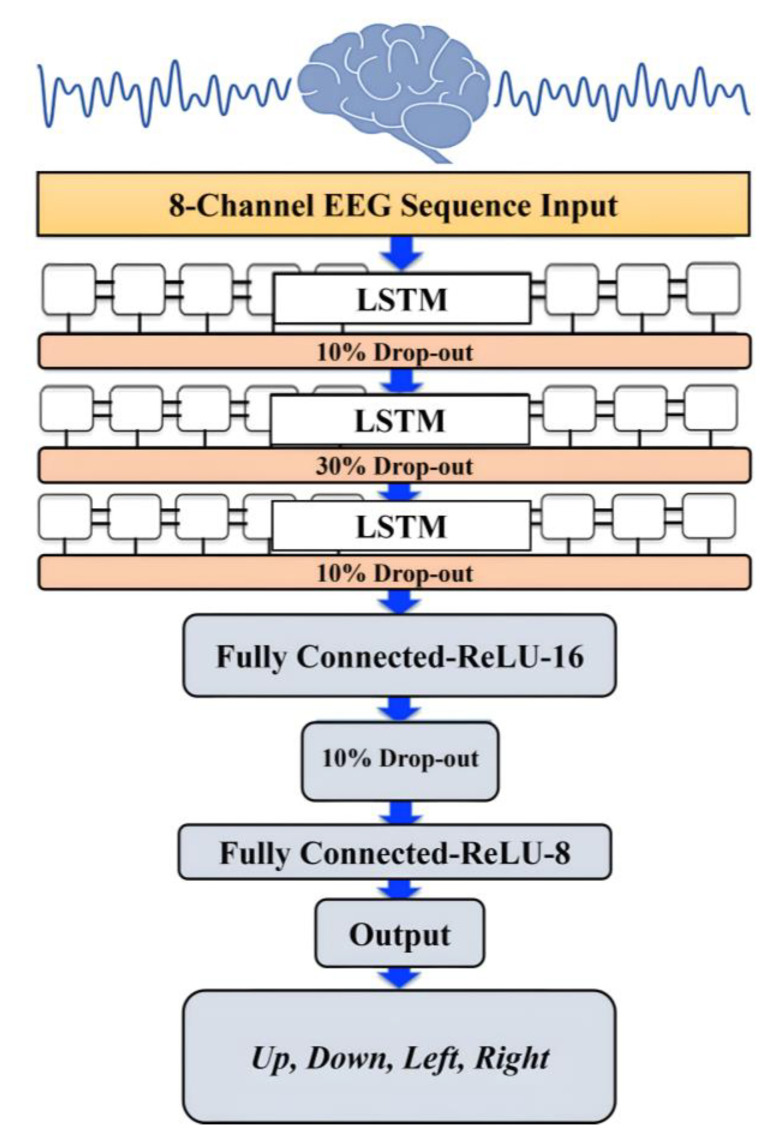
The architecture of the LSTM model.

The performance of the LSTM network depends on several hyper-parameters, such as the network size, initial learning rate, learning rate schedule, and L2 regularization. The initial learning rate was set at 0.019 and scheduled with a 0.017 reduction ratio every 176 epoch. To prevent or reduce overfitting in the training process, we considered dropout ratios of 0.1, 0.3, and 0.1 for the training parameters in the LSTM neural network layers. The dropout layers randomly set 10%, 30%, and 10% of the training parameters to zero in the first, second, and third LSTM layers, respectively. Another technique was used to overcome the overfitting in the learning process and for a smoother training process, which is the L2 regularization. The L2 regularization is the most common type of all regularization techniques and is also commonly known as weight decay or ride regression. Figure 6 illustrates the architecture of the designed LSTM model.

The mathematical form of this regularization technique can be summarized in the following two equations:

(2) $$\Omega (w)=\|W{\|}_{2}^{2}=\sum_{i}\sum_{j}w_{ij}^{2}$$

(3) $$L(W)=\frac{\alpha }{2}\|W{\|}_{2}^{2}+L(W)=\frac{\alpha }{2}\sum_{i}\sum_{j}w_{ij}^{2}+L(W)$$

During the *L2* regularization, the loss function of the neural network is expressed by a purported regularization term, which is called Ω in (2). *W* is the weight vector, and the regularization function is Ω*(*w*)*. The regularization term Ω is defined as the *L2* norm of the weight matrices (*W*), which is the summation of all squared weight values of a weight matrix. The regularization term is weighted by the scalar α divided by two and added to the regular loss function *L(W)* in (3). The scalar α is sometimes called the regularization coefficient (initial value was set to 0.0001) and is a supplementary hyperparameter introduced into the neural network, which determines how much the model is being regularized. The network ended with two fully connected and SoftMax output layers, with the number of class labels equal to the desired number of the four outputs. Two fully connected layers and one dropout layer with a 0.1 dropout ratio were added after the output of the LSTM hidden units. These two fully connected layers consisted of 16 and 8 nodes and used ReLU activation functions, and these two layers computed the weighted sum of the inputs and passed the output to the final output layer.

## 3. Results

**Figure 7:**
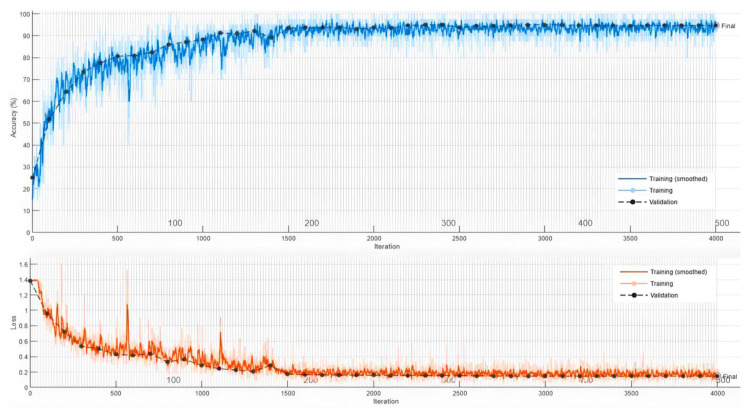
The data validation and loss curves during the training process of the LSTM model using MATLAB.

Using the eight-channel EEG headset enabled us to design a minimally computationally-intensive algorithm to distinguish between four imagined speech commands. Moreover, using the wavelet scattering transformation improved the simplicity of the EEG dataset by extracting features from each channel and reducing the dimension of the EEG feature matrix. The feature matrix was calculated for each recording of the four imagined speech commands. Using the feature matrices to train the LSTM model improved the learning process and its the execution time. In [20], a 64-channel EEG headset was used to record 8 min of imagined tones for each of the 14 participants in the study. Mixed visual and auditory stimuli were used, and a maximum classification accuracy of 80.1% was achieved in classifying four EEG-based imagined tones. We used a low cost 8-channel EEG-headset to record a total of 100 min for each of the four participated subjects. Using the auditory stimuli by asking a question to the participants showed that greater accuracy could be achieved in an offline BCI system to classify an imagined speech. An accuracy of 92.50% was achieved when testing the resulting LSTM model on the remaining 20% of the normalized and filtered EEG dataset. The results were achieved with the utilization of the adaptive moment estimation (Adam) optimizer. The Adam optimizer is a method for calculating the adaptive learning rate for each of the hyperparameters of the LTSM-RNN model. We achieved 92.50% after training the LSTM-RNN model on 80% of the recorded EEG dataset with 500 max epochs and 40 for mini-batch size. Figure 7 illustrates the data validation and loss curves during the training process of the LSTM model using MATLAB.

**Figure 8:**
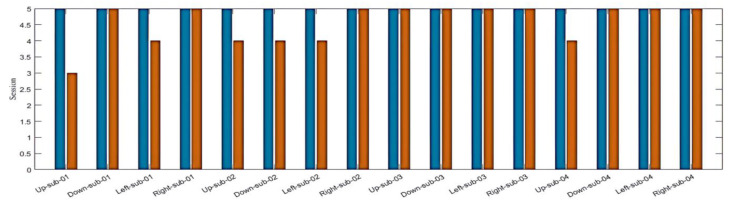
The performance of the designed LSTM model. The wrong predicted commands (red bars) were only 6 out of 80 (5 recordings per participant) for all participants, which leads to 92.50% accuracy in the overall prediction of the designed LSTM model.

By employing the LSTM model, we could distinguish between four different imagined speech-based commands. For each command, 20 recordings were used for the testing stage, and the nominal values (0, 0.5, 0.7, and 1.0) were assigned for each command as an output value, respectively. The output value of (0), representing the command Up, predicted (16/20) of the expected outputs and achieved a classification accuracy of80%. The output values of (0.5) and (0.7), which represent the commands down and left, predicted (19/20) of the expected outputs and achieved a classification accuracy of 95%. Meanwhile, the output value of (1.0), which represents the command right, predicted (20/20) of the expected outputs and achieved a classification accuracy of 100%. We calculated the 92.50% overall classification accuracy from averaging the (80%, 95%, 95%, 100%) results from each imagined speech command. Figure 8 illustrates the classification accuracy of the designed LSTM model.

**Figure 9:**
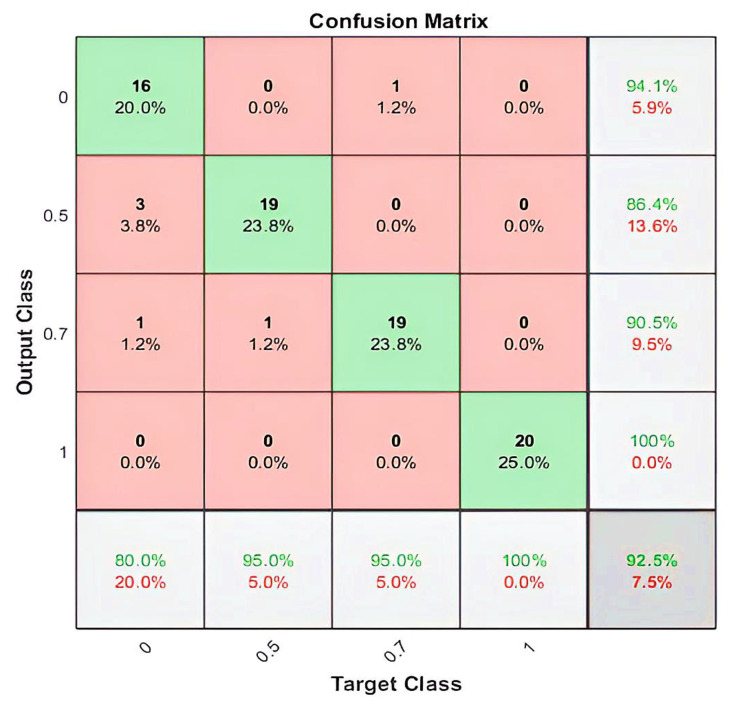
The confusion matrix for the classification of the four imagined speech commands. The rows represent the predicted class and the columns represent the true class. The diagonal (green) cells correspond to observations that are correctly classified. The off-diagonal (red) cells correspond to incorrectly classified observations. Both the number of observations and the percentage of the total number of observations are shown in each cell. The column on the far right of the plot shows the percentages of all the samples predicted to belong to each class that are correctly and incorrectly classified. The row at the bottom of the plot shows the percentages of all the samples belonging to each class that are correctly and incorrectly classified.

Figure 9 illustrates the number and percentage of correct classifications by the trained LSTM network.

The column on the far right of the plot shows the percentages of all the examples predicted to belong to each class that are correctly and incorrectly classified. These metrics are often called the precision (or positive predictive value) and false discovery rate, respectively. The row at the bottom of the plot shows the percentages of all the examples belonging to each class that are correctly and incorrectly classified. These metrics are often called the recall (or true positive rate) and false negative rate, respectively. The cell in the bottom right of the plot shows the overall accuracy.

For better evaluation of the performance of the trained LSTM model, the classified dataset was categorized into true positive, true negative, false positive, and false negative. The number of true positive and true negative are the number of classes that were correctly classified. Numbers of false positive and false negative are the numbers of classes that were misclassified. The state-of-the-art metrics for classification are accuracy, precision, recall, and F-score. The recall, sometimes called sensitivity, estimates the ratio of true positive from the total number of true positive and false negative. Precision estimates the ratio of true positive from the total number of true positive and false negative. The F-score estimates the average between the recall and precision. Using the above confusion matrix, we calculated all the three metrics, and we obtained 92.74%, 92.50%, and 92.62% for precision, recall, and F1-score, respectively.

## 4. Discussion

Although the overall accuracy of classifying the imagined speech for the designed BCI system is considered excellent, one of the commands still needs improvements to show a higher accuracy compared with the other three commands. For each of the 100 recordings, the participants imagined saying each of the individual four commands. In [24], the participants were instructed to keep performing speech imagery for up to 14 s, but they only responded when they heard a beep until the visual cue disappeared. In [25], in each recording, a visual cue was used and the participants were instructed to perform 30 s of repeating six different words using speech imagery. Unlike the recording scenario in [24,25], we did not use any cues during the 60 s chosen response time. In fact, our participants were instructed to keep repeating the speech imagery for a single command in each recording. The first command, up, was always the first to be imagined and the results showed that up has the highest prediction error in all the participants. The reason might be because the participant’s brain adapts to the speech-imagining process gradually. At the beginning of the recording, a participant might not have been focused enough to produce a good EEG signal while imagining saying a command. Another reason might be that the timing to present the question was not enough to generate the best EEG signal, especially at the beginning of the recording when the question was immediately announced as soon as the recording had started. Another limitation is related to the participants, who were all healthy subjects without any challenges in normal speech or language production. 

Although the recorded EEG dataset has a potential flaw, we still have an excellent performing LSTM imagined speech classification model that can be used to decode our brain thoughts. We used audio cues to stimulate the brain by asking a question to the participants and let the person imagine the response, unlike [27,29] where visual cues were used. The resulting LSTM model can be converted to a C++ or Python code using MATLAB code generation and uploaded to a microcontroller to be tested in real-time. 

## 5. Conclusions

A BCI system is particularly beneficial if it can be converted into an operational and practical real-time system. Although the offline BCI approach allows the researchers to use computationally expensive algorithms for processing the EEG datasets, it is applicable only in a research environment. This research provided insights towards using a low-cost EEG headset with a low number of channels to develop a reliable BCI system using a minimized computing for optimum learning process. We accomplished the resulting imagined speech classification model by employing the LSTM neural architecture in the learning and classification process. We placed the EEG sensors on carefully selected spots on the scalp to demonstrate that we could obtain classifiable EEG data with fewer sensors. By employing wavelet scattering transformation, the classified EEG signals showed the possibility of building a reliable BCI to translate brain thoughts to speech and help physically challenged people to improve the quality of their lives. All the testing and training stages were implemented offline without any online testing or execution. Future work is planned to implement and test an online BCI system using MATLAB/Simulink and g.tec Unicorn Hybrid Black+ headset. 

## 6. Future Work

Further deep learning and filtration techniques will be implemented on the EEG dataset to improve the classification accuracy. We obtained a promising preliminary result with the support vector machine (SVM) classification model. Online testing for the resulting classification model is planned to be implemented using MATLAB Simulink for better evaluation of the classification performance in real-time.

## Figures and Tables

**Table 1:** Participants Information.

Participant	Gender	Age	Native Language
sub-01	Male	56	English
sub-02	Female	20	English
sub-03	Male	29	English
sub-04	Female	26	English

## Data Availability

The datasets used and/or analyzed during the current study are available from the corresponding author upon approved written requests.
